# Delaware

**Published:** 1897-08

**Authors:** 


*Delaware.* The present Legislature has passed a law providing for the payment of eight dollars for each animal dying from anthrax, in order to provide for the proper and safe burial of the carcasses.

